# Risk Factors for Prolonged Pleural Effusion Following Total Cavopulmonary Connection Surgery: 9 Years' Experience at Fuwai Hospital

**DOI:** 10.3389/fped.2019.00456

**Published:** 2019-11-07

**Authors:** Qipeng Luo, Wei Zhao, Zhanhao Su, Yiwei Liu, Yuan Jia, Liang Zhang, Hongbai Wang, Yinan Li, Xie Wu, Shoujun Li, Fuxia Yan

**Affiliations:** ^1^Department of Anesthesiology, Fuwai Hospital, National Center of Cardiovascular Diseases, Chinese Academy of Medical Sciences and Peking Union Medical College, Beijing, China; ^2^Department of Information, Fuwai Hospital, National Center of Cardiovascular Diseases, Chinese Academy of Medical Sciences and Peking Union Medical College, Beijing, China; ^3^Center for Pediatric Cardiac Surgery, Fuwai Hospital, National Center of Cardiovascular Diseases, Chinese Academy of Medical Sciences and Peking Union Medical College, Beijing, China; ^4^Department of Anesthesiology, Chongqing Hospital of Traditional Chinese Medicine, Chongqing, China

**Keywords:** prolonged pleural effusion, congenital heart disease, total cavopulmonary connection, fenestration, chylothorax

## Abstract

**Background:** Prolonged pleural effusion (PPE) contributes to adverse outcomes after total cavopulmonary connection (TCPC) completion. We aimed to identify risk factors for PPE following TCPC surgery.

**Methods:** We studied a retrospective cohort of 525 who undergoing TCPC surgery from 2010 to 2019. We defined PPE as the duration of pleural effusion exceeding 14 days. Logistic regression was applied to identify risk factors for PPE and Cox regression was used to identify risk factors for predicting the duration of pleural effusion. The impacts of PPE on the short-term outcomes were evaluated.

**Results:** The rate of PPE was 27.4% in our study and independent risk factors for PPE included: young age, no fenestration, low postoperative total protein, prolonged mechanical ventilation and chylothorax. These predictors were also achieved in the Cox regression for predicting the duration of pleural effusion. The applicability of the model was acceptable in different subgroups, which derived from the total cohort. Patients with PPE were associated with more renal replacement treatment, longer length of ICU and hospital stay, more hospitalization costs and a higher rate of in-hospital mortality.

**Conclusions:** PPE in our study occurs at a relatively lower rate than that reported in previous studies and patients with PPE was associated with higher rate of in-hospital mortality when compared to patients without PPE. Young age, no fenestration, low postoperative total protein, prolonged mechanical ventilation, and chylothorax were identified as independent risk factors to predict PPE. A preventive strategy that targets the identified risk factors to reduce the incidence of PPE following TCPC surgery could be beneficial for in-hospital outcomes, and the model needs further validation before its application.

## Introduction

Total cavopulmonary connection (TCPC) is one of the most frequently performed procedures in children with complex congenital heart disease. Currently, there are about 22,000 and 50,000 cases of functionally univentricular heart disease in Europe and the US, respectively (1). The early and late mortality after TCPC surgery was about 2.9 and 3.4% (2, 3). Although significant advances in surgical techniques had been made in the past years, numerous challenges remain to care for these patients. Following TCPC surgery, various short-term morbidities may occur, such as prolonged pleural effusion (PPE), arrhythmias, acute kidney injury, etc., which are associated with adverse in-hospital and long-term outcomes.

Following cardiac surgery, pleural effusion is a common complication with potential impact on outcomes. In previous studies, pleural effusion has been shown a notable risk factor for prolonged ICU stay and long-term morbidities (4, 5), and PPE also independently increases the risk of early and late deaths (6, 7). Despite several risk factors for PPE were reported in the current literature, including cardiopulmonary bypass longer than 120 min, heterotaxy, no fenestration, increased pulmonary artery pressure, pulmonary atresia, low preoperative peripheral oxygen saturation, and postoperative infection (8–11). However, the studies did not include early postoperative biomarkers, which represent functions of main organs after the surgery, and the injuries of the main organs might influence the duration of pleural effusion. In addition, the studies might not be applicable to a Chinese setting, in which the age at operation is older than that in the developed countries.

To address the current knowledge gap, we aim to identify risk factors for PPE and to investigate the consequences of PPE following TCPC surgery.

## Materials and Methods

### Study Population

From July 2010 to May 2019, patients who received TCPC completion under cardiopulmonary bypass (CPB) at Fuwai Hospital were, respectively analyzed. Patients who died within 14 days after the surgery were excluded. The study was approved by the Research Ethics Board of Fuwai Hospital and all data were retrieved from electronic medical records.

### Definition of Outcome Variables

PPE was defined as the postoperative duration of pleural effusion exceeding 14 days.

### Data Collection and Variable Definition

All possible predicting variables were included on the basis of clinical judgment, literature review and availability in our hospital. The preoperative baseline data were collected: age at operation, weight, gender, body surface area, peripheral oxygen saturation (SpO2) inhaling air when admission to the hospital, preoperative pulmonary artery pressure, prior Glenn, prior B-T shunt, heterotaxy syndrome diagnosis, original anatomy and ventricle morphology. The following pre- and postoperative blood laboratory parameters were collected: count of white blood cell, neutrophil, lymphocyte, monocyte and platelet, levels of hemoglobin, prothrombin time, total protein, albumin, alanine aminotransferase, aspartate aminotransferase, alkaline phosphatase, total bilirubin, serum creatinine, isoenzyme of creatine kinase-MB, lactate dehydrogenase and high-sensitivity C-reactive protein. The following preoperative echocardiography variables were collected: main ventricle ejection fraction, main ventricle end-diastolic diameter z-score, atrioventricular valve regurgitation and Nakata index. Intraoperative variables which reflected the status during the surgery included senior surgeons who performed TCPC procedure over 10 cases per year averagely, preoperative SpO2 inhaling 100% oxygen after intubation, CPB time, aortic cross-clamp time, creation of fenestration, internal tunnel procedure, concomitant atrioventricular valve surgery, minimal temperature during CPB, maximum vasoactive-Inotropic score (VIS) during surgery, postoperative SpO2 inhaling 50-60% oxygen after surgery and intraoperative fluid balance. The following postoperative variables were also collected: maximal lactic acid within 6 h after surgery, cumulative fluid balance within 72 h after surgery and postoperative pulmonary artery pressure when admission to ICU.

The preoperative variables were measured at the closest time before surgery and postoperative variables were the first measurements when admitted to the ICU. The diagnosis of chylothorax was reached by high concentration of triglycerides (110 mg/dL or > serum triglycerides) and lymphocytes (80% of cells) in the pleural effusion at the postoperative day 5–7. Renal replacement treatment included peritoneal dialysis and blood filtration.

Fluid balance (mL/kg) was calculated: Fluid balance = [(amount of crystalloids + colloids + packed red cell + plasma + platelets + enteral nutrition) – (blood loss + urine output + gastrointestinal losses + drain losses + dialysis or ultrafiltrate)]/weight. Z-scores indexed to body surface area were applied when calculating left ventricular end-diastolic diameter z-score. Estimated glomerular filtration rate (eGFR) was computed: eGFR = 0.413^*^height (cm) / serum creatinine (SCr) level (mg/dL) (12). VIS was calculated: dopamine (μg/kg/min) + dobutamine (μg/kg/min) + 10 ^*^ milrinone (μg/kg/min) + 50 ^*^ levosimendan (μg/kg/min) + 100 ^*^ epinephrine (μg/kg/min) + 100 ^*^ norepinephrine (μg/kg/min) + 10,000 ^*^ vasopressin (U/kg/min) (13).

### Perioperative Management

Patients before TCPC were administrated with normal diet. CPB with or without aortic cross-clamping was chosen if necessary and ultrafiltration was used in all patients after CPB. All patients received extracardiac TCPC surgery and the conduit size was selected according the age and weight at the operation. All patients were admitted to the ICU after TCPC surgery. The biochemical testing for chylothorax was routinely examined in our hospital on postoperative day 5–7. The decision of chest tube removal was made if the drainage of pleural effusion <2 mL/kg/day/tube. Conventional anticoagulant therapy was started at night on the same day as the operation depending on coagulation function. In patients without chylothorax, low-fat diet was given after TCPC until chest tube removal; in those with chylothorax, low-fat diet was given until 6 weeks after discharge from hospital. Other managements were based on the routine practice of Fuwai Hospital.

### Statistical Analysis

Missing data were imputed using the R package “missForest” with random forest algorithm. Distributions of the recorded data, imputed data, and mixed data were compared to assess the impact of the imputation process on the original data. Logistic regression using backward elimination method was applied to identify the predictors for PPE and Cox regression with backward elimination method was used for predicting the duration of pleural effusion. Variables with *p* ≤ 0.15 in univariate analysis were put into multivariate logistic and Cox models, and multicollinearity was evaluated before the variables were put into the multivariate logistic or Cox analysis. Ordinal logistic regression was applied to examine the relationship between different groups of pleural effusion duration (<14, 14–21, >21 days) and postoperative outcomes. Six subgroups were used to identify the application ability of the PPE model in different population. The calibration and discrimination ability of the model were assessed by calibration curve, Hosmer-Lemeshow goodness-of-fit test (HL test) and receiver operating characteristic curve (ROC). *P* ≤ 0.05 was deemed statistically significant. All analyses were performed in R software (version 3.6.0).

## Results

### Missing Data

There were no significant differences among the distributions of the recorded data, imputed data and mixed data. The rate of missing data in this study ranged from 0.2% to 5.3%. In total 429 out of 525 records (82%) were complete cases. Among the 5 variables included in the logistic model, no variables had missing values (Table 1).

**Table 1:** Missing measurements.

**Variables**	**Total cohort (*****N*** **= 525)**	
	**No**.	**Percentage (%)**
Nakata index	5	0.9
Preoperative pulmonary arterial pressure	22	4.1
Ventricular ejection fraction	1	0.2
Ventricular end-diastolic diameter z-score	12	2.3
Preoperative prothrombin time	2	0.4
Preoperative alanine aminotransferase	1	0.2
Preoperative aspartate aminotransferase	1	0.2
Preoperative alkaline phosphatase	16	3
Preoperative total bilirubin	14	2.6
Preoperative lactate dehydrogenase	2	0.4
Preoperative high-sensitivity C-reactive protein	16	3
Postoperative lactic acid	10	1.9
Postoperative prothrombin time	25	4.7
Postoperative alkaline phosphatase	27	5.1
Postoperative total bilirubin	27	5.1
Postoperative isoenzyme of creatine kinase-MB	2	0.4
Postoperative lactate dehydrogenase	2	0.4
Postoperative high-sensitivity C-reactive protein	28	5.3

### Characteristics of the Study Population

A total of 531 patients received TCPC surgery from July 2010 to May 2019 at our hospital. Six patients died within 14 days after the surgery and were excluded. A total of 525 patients finally included in the study.

The median and quartile of the age in the total cohort were 6 (4.2, 11) years old. Among 525 patients, 6.1% patients had the diagnosis of heterotaxy syndrome, the percentages of right ventricle morphology and left ventricle morphology were 39.6 and 39%, respectively, the diagnosis of unbalanced atrioventricular septal defect, tricuspid atresia and unbalanced double outlet right ventricle account for 67.8% of the disease, 62.7% patients had the history of prior Glenn, 45.1% patients created a fenestration and 10.1% patents performed a concomitant atrioventricular valve surgery. All patients received CPB and 66.7% patients underwent aortic cross-clamping. The CPB and aortic cross-clamp time were 128 ± 67 min and 27.6 ± 46 min, respectively. The perioperative characteristics of the study patients with *p* < 0.15 in univariate analysis are displayed in Table 2 and the anatomy characteristics of the study patients are shown in Table 3.

**Table 2:** The perioperative characteristics of the study patients with *p* < 0.15 in univariate analysis.

**Variables**	**Non-PPE** **(*n* = 381)**	**PPE** **(*n* = 144)**	***P*-value**	**Overall** **(*n* = 525)**
Age at operation (years)	7.0 (4.3, 13.0)	5.8 (4.0, 8.0)	<0.001	6.0 (4.2, 11.0)
Weight (kg)	19.5 (16.0, 32.0)	18.0 (15.0, 22.5)	0.004	19.0 (15.5, 29.0)
Body surface area (m^2^)	0.79 (0.67, 1.12)	0.74 (0.65, 0.88)	0.003	0.77 (0.66, 1.05)
Glenn surgery history (yes)	230 (60.4%)	99 (68.8%)	0.09	329 (62.7%)
Surgeons (senior)	228 (59.8%)	98 (68.1%)	0.08	326 (62.1%)
Original anatomy			0.047	
Unbalanced atrioventricular septal defect	100 (26.2%)	44 (30.6%)		144 (27.4%)
Tricuspid atresia	92 (24.1%)	21 (14.6%)		113 (21.5%)
Unbalanced double outlet right ventricle	71 (18.6%)	28 (19.4%)		99 (18.9%)
Double inlet ventricle	40 (10.5%)	8 (5.6%)		48 (9.1%)
Pulmonary atresia	24 (6.3%)	18 (12.5%)		42 (8.0%)
Complete transposition of great arteries	18 (4.7%)	8 (5.6%)		26 (5.0%)
CCTGA	26 (6.8%)	13 (9.0%)		39 (7.4%)
Others	10 (2.6%)	4 (2.8%)		14 (2.7%)
Main ventricle ejection fraction ≤55%	48 (12.6%)	22 (15.3%)	0.148	70 (13.3%)
Preoperative lymphocyte (× 10^9^/L)	3.58 (2.78, 4.69)	3.77 (2.77, 4.92)	0.13	3.60 (2.78, 4.76)
Preoperative hemoglobin (g/dL)	18.0 (16.2, 20.3)	17.0 (15.9, 19.3)	0.002	17.8 (16.0, 20.0)
Preoperative platelet (× 10^10^/L)	23.1 (17.5, 28.7)	25.3 (21.1, 31.3)	<0.001	24.0 (18.3, 29.1)
Preoperative prothrombin time (s)	14.4 (13.9, 15.2)	14.2 (13.7, 14.8)	0.006	14.4 (13.8, 15.2)
Preoperative alamine aminotransferase (U/L)	15 (12, 20)	14 (11, 18)	0.005	15 (11 20)
Preoperative aspartate aminotransferase (U/L)	25 (21, 30)	27 (23, 33)	0.007	26 (22, 31)
Preoperative CKMB (U/L)	23 (19, 30)	26 (20, 33)	0.02	24 (19, 31)
Creaction of fenestraion (yes)	193 (50.7%)	44 (30.6%)	<0.001	237 (45.1%)
Maximal VIS during surgery	11.0 (6.0, 14.0)	11.0 (9.0, 17.0)	0.005	11.0 (7.0, 15.0)
Intraoperative fluid balance (mL/kg)	6.2 (−9.0, 23.6)	11.6 (−6.7, 30.6)	0.05	8.9 (−8.9, 25.4)
Lactic acid (mmol/L)	1.9 (1.4, 2.9)	2.0 (1.3, 3.1)	0.15	1.9 (1.4, 2.9)
Postoperative lymphocyte (× 10^9^/L)	0.99 (0.69, 1.49)	1.2 (0.86, 1.61)	0.004	1.05 (0.73, 1.54)
Postoperative hemoglobin (g/dL)	14.2 (12.6, 16.1)	13.4 (11.9, 15.3)	0.01	14.0 (12.4, 15.9)
Postoperative platelet (× 10^10^/L)	17.2 (12.9, 23.2)	18.8 (14.3, 24.3)	0.154	17.5 (13.5, 23.3)
Postoperative prothrombin time (s)	15.8 (14.9, 17.0)	15.9 (15.0, 18.0)	0.1	15.8 (15.0, 17.2)
Postoperative total protein (g/L)	62.6 ± 7.74	59.9 ± 8.53	0.001	61.8 ± 8.04
Postoperative albumin (g/L)	41.8 ± 5.63	40.7 ± 6.19	0.05	41.5 ± 5.81
Postoperative alamine aminotransferase (U/L)	20 (16, 27)	21 (16, 41)	0.11	20 (16, 28)
Postoperative aspartate aminotransferase (U/L)	50 (39, 74)	59 (45, 100)	<0.001	53 (40, 78)
Postoperative TBIL (≥38 μmol/L)	232 (60.9%)	76 (52.8%)	0.12	308 (58.7%)
Postoperative lactate dehydrogenase (U/L)	403 (334, 508)	437 (338, 576)	0.04	411 (335, 528)
Postoperative high-sensitivity C-reactive protein (mg/L)	13.2 (12.2, 14.0)	12.9 (11.6, 13.8)	0.014	13.1 (12.0, 14.0)
Postoperative fluid balance (mL/kg)	−29.2 (−55.2, −10.9)	−17.0 (−33.8, 3.7)	<0.001	−25.9 (−50.8, −5.4)
Postoperative mechanical ventilation (≥48 h)	37 (9.7%)	32 (22.2%)	<0.001	69 (13.1%)
Chylothorax (yes)	151 (39.6%)	95 (66.0%)	<0.001	246 (46.9%)

*PPE, prolonged pleural effusion; CCTGA, congenitally corrected transposition of the great arteries; CKMB, isoenzyme of creatine kinase-MB; VIS, maximum vasoactive-Inotropic score during surgery; TBIL, total bilirubin*.

**Table 3:** The anatomy characteristics of the study patients.

**Variables**	**Non-PPE** **(*n* = 381)**	**PPE** **(*n* = 144)**	***P*-value**	**Overall** **(*n* = 525)**
Heterotaxy syndrome	21 (5.5%)	11 (7.6%)	0.417	32 (6.1%)
Ventricle morphology			0.160	
Intermediated	80 (21.0%)	32 (22.2%)		112 (21.3%)
Left	158 (41.5%)	47 (32.6%)		205 (39.0%)
Right	143 (37.5%)	65 (45.1%)		208 (39.6%)
Original anatomy			0.047	
Unbalanced atrioventricular septal defect	100 (26.2%)	44 (30.6%)		144 (27.4%)
Tricuspid atresia	92 (24.1%)	21 (14.6%)		113 (21.5%)
Unbalanced double outlet right ventricle	71 (18.6%)	28 (19.4%)		99 (18.9%)
Double inlet ventricle	40 (10.5%)	8 (5.6%)		48 (9.1%)
Pulmonary atresia	24 (6.3%)	18 (12.5%)		42 (8.0%)
Complete transposition of great arteries	18 (4.7%)	8 (5.6%)		26 (5.0%)
Congenitally corrected transposition of the great arteries	26 (6.8%)	13 (9.0%)		39 (7.4%)
Others	10 (2.6%)	4 (2.8%)		14 (2.7%)

*PPE, prolonged pleural effusion*.

### Logistic Regression for Predicting the PPE

Among the 64 variables, 32 variables had *p* ≤ 0.15 in the univariate logistic analysis and the five variables were the independent predictors in the multivariate logistic analysis. The five independent risk factors were young age at operation, no fenestration, low postoperative total protein level, prolonged mechanical ventilation and chylothorax. The odd ratios of the logistic analysis are shown in Table 4.

**Table 4:** Univariate and multivariate logistic analysis odds ratios for prolonged pleural effusion.

**Variables**	**Univariate analysis**		**Multivariate analysis**	
	**OR (95%CI)**	**p**	**OR (95%CI)**	***p***
Age at operation (years)	**0.91 (0.88, 0.95)**	**0.001**	**0.91 (0.87, 0.95)**	**<0.001**
Weight (kg)	0.97 (0.95, 0.99)	0.001		
Body surface area (m^2^)	0.29 (0.15, 0.59)	0.001		
Glenn surgery history (yes vs. no)	1.44 (0.96, 2.17)	0.074		
Surgeons (senior vs. junior)	1.43 (0.95, 2.15)	0.081		
Original anatomy (Unbalanced AVSD as Ref.)				
Tricuspid atresia	0.52 (0.29, 0.94)	0.03		
Unbalanced DORV	0.9 (0.51, 1.57)	0.703		
Double inlet ventricle	0.45 (0.2, 1.05)	0.065		
Pulmonary atresia	1.7 (0.84, 3.46)	0.139		
TGA	1.01 (0.41, 2.5)	0.983		
CCTGA	1.14 (0.53, 2.42)	0.74		
Others	0.91 (0.27, 3.06)	0.878		
Main ventricle ejection fraction (>55% vs. ≤55%)	0.8 (0.46, 1.18)	0.15		
Preoperative lymphocyte (× 10^9^/L)	1.1 (0.98, 1.24)	0.102		
Preoperative hemoglobin (g/dL)	0.9 (0.84, 0.97)	0.003		
Preoperative platelet (× 10^10^/L)	1.04 (1.02, 1.06)	0.001		
Preoperative prothrombin time (s)	0.89 (0.77, 1.02)	0.084		
Preoperative alamine aminotransferase (U/L)	0.97 (0.95, 0.998)	0.028		
Preoperative aspartate aminotransferase (U/L)	1.03 (1.01, 1.05)	0.01		
Preoperative CKMB (U/L)	1.02 (1.01, 1.03)	0.038		
Creaction of fenestraion	**0.43 (0.29, 0.64)**	**0.001**	**0.51 (0.33, 0.79)**	**0.003**
Maximal VIS during surgery	1.03 (1.01, 1.05)	0.027		
Intraoperative fluid balance (mL/kg)	1.01 (0.98, 1.013)	0.075		
Lactic acid (mmol/L)	1.1 (0.98, 1.25)	0.113		
Postoperative lymphocyte (× 10^9^/L)	1.44 (1.11, 1.87)	0.007		
Postoperative hemoglobin (g/dL)	0.92 (0.85, 0.99)	0.021		
Postoperative platelet (× 10^10^/L)	1.01 (1.002, 1.03)	0.084		
Postoperative prothrombin time (s)	1.09 (1.02, 1.17)	0.005		
Postoperative total protein (g/L)	**0.96 (0.94, 0.98)**	**0.001**	**0.96 (0.93, 0.99)**	**0.024**
Postoperative albumin (g/L)	0.97 (0.94, 0.997)	0.044		
Postoperative alamine aminotransferase (U/L)	1.0008 (1.0002, 1.0015)	0.003		
Postoperative aspartate aminotransferase (U/L)	1.0007 (1.0002, 1.0013)	0.001		
Postoperative TBIL (≥38 vs. <38 μmol/L)	0.72 (0.49, 1.06)	0.093		
Postoperative lactate dehydrogenase (U/L)	1.0006 (1.0002, 1.001)	0.002		
Postoperative high-sensitivity C-reactive protein (mg/L)	0.95 (0.89, 1.02)	0.137		
Postoperative fluid balance (mL/kg)	1.0036 (0.9993, 1.008)	0.072		
Postoperative mechanical ventilation (≥48 vs. <48 h)	**2.66 (1.58, 4.46)**	**0.001**	**2.52 (1.35, 4.73)**	**0.004**
Chylothorax (yes vs. no)	**2.95 (1.98, 4.41)**	**0.001**	**2.6 (1.7, 3.98)**	**<0.001**

*OR, odds ratios; AVSD, atrioventricular septal defect; DORV, double outlet right ventricle; TGA, complete transposition of great arteries; CCTGA, congenitally corrected transposition of the great arteries; CKMB, isoenzyme of creatine kinase-MB; VIS, maximum vasoactive-Inotropic score during surgery; TBIL, total bilirubin. Bold values mean statistical p < 0.05*.

### Cox Regression for Predicting the Duration of Pleural Effusion

Thirty-three variables with *p* ≤ 0.15 in the univariate Cox regression were entered into the multivariate Cox regression and seven variables were identified as independent factors for predicting the duration of pleural effusion. The seven independent predictors included age at operation, main ventricle ejection fraction, creation of fenestration, postoperative levels of total protein and alkaline phosphatase, postoperative mechanical ventilation and chylothorax. The hazard ratios of the logistic analysis are shown in Table 5.

**Table 5:** Univariate and multivariate Cox analysis hazard ratios for duration of pleural effusions.

**Variables**	**Univariate analysis**		**Multivariate analysis**	
	**HR (95%CI)**	***p***	**HR (95%CI)**	***p***
Age at operation (yrs)	**0.96 (0.95, 0.97)**	**<0.001**	**0.95 (0.93, 0.96)**	**<0.001**
Weight (kg)	0.98 (0.98, 0.99)	<0.001		
Body surface area (m^2^)	0.49 (0.37, 0.64)	<0.001		
Admission SpO2 (≥80 vs. <80%)	1.14 (0.95, 1.36)	0.147		
Glenn surgery history (yse vs. no)	1.45 (0.94, 2.22)	0.094		
Original anatomy (Unbalanced AVSD as Ref.)				
Tricuspid atresia	0.83 (0.65, 1.06)	0.140		
Unbalanced DORV	1.09 (0.84, 1.41)	0.52		
Double inlet ventricle	0.68 (0.49, 0.95)	0.02		
Pulmonary atresia	1.43 (1.001, 2.04)	0.049		
TGA	1.02 (0.67, 1.54)	0.95		
CCTGA	0.93 (0.65, 1.33)	0.7		
Others	0.87 (0.5, 1.51)	0.62		
Main ventricle ejection fraction (>55% vs. ≤55%)	**0.91 (0.71, 1.18)**	**0.14**	**0.69 (0.54, 0.90)**	**0.006**
Preoperative hemoglobin (g/dL)	0.95 (0.92, 0.98)	0.001		
Preoperative platelet (× 10^10^L)	1.02 (1.01, 1.03)	0.000		
Preoperative prothrombin time (s)	0.95 (0.91, 0.99)	0.018		
Preoperative alamine aminotransferase (U/L)	0.99 (0.98, 0.9995)	0.038		
Preoperative aspartate aminotransferase (U/L)	1.02 (1.01, 1.03)	0.003		
Preoperative TBIL (≥38 vs. <38 μmol/L)	0.71 (0.48, 1.04)	0.076		
Preoperative isoenzyme of creatine kinase-MB (U/L)	1.01 (1.001, 1.02)	0.019		
CPB time (min)	0.999 (0.998, 1.0003)	0.142		
Clamp time (min)	0.998 (0.996, 1.0001)	0.058		
Creaction of fenestraion	**0.59 (0.49, 0.71)**	**0.000**	**0.62 (0.52, 0.74)**	**<0.001**
Maximal VIS during surgery	1.01 (0.999, 1.023)	0.061		
Intraoperative fluid balance (mL/kg)	1.002 (0.999, 1.005)	0.146		
Postoperative pulmonary artery pressure (mmHg)	1.02 (0.99, 1.07)	0.141		
Postoperative lymphocyte (× 10^9^/L)	1.2 (1.06, 1.36)	0.005		
Postoperative hemoglobin (g/dL)	0.96 (0.93, 0.99)	0.006		
Postoperative total protein (g/L)	**0.98 (0.97, 0.995)**	**0.006**	**0.98 (0.97, 0.99)**	**0.038**
Postoperative albumin (g/L)	0.99 (0.97, 1.003)	0.114		
Postoperative alamine aminotransferase (U/L)	1.0002 (1.0001, 1.001)	0.111		
Postoperative aspartate aminotransferase (U/L)	1.0002 (1.0001, 1.001)	0.076		
Postoperative alkaline phosphatase	**0.99 (0.98, 1.002)**	**0.12**	**0.994 (0.98, 0.998)**	**0.037**
Postoperative TBIL (≥38 vs. <38 μmol/L)	0.84 (0.7, 0.996)	0.045		
Postoperative lactate dehydrogenase (U/L)	1.0002 (1.0001, 1.001)	0.030		
Postoperative high-sensitivity C-reactive protein (mg/L)	0.97 (0.95, 1.002)	0.064		
Postoperative fluid balance (mL/kg)	1.001 (1.0001, 1.002)	0.032		
Postoperative mechanical ventilation (≥48 vs. <48 h)	**1.5 (1.17, 1.94)**	**0.002**	**1.57 (1.17, 2.09)**	**0.002**
Chylothorax (yes vs. no)	**1.88 (1.57, 2.24)**	**<0.001**	**1.73 (1.44, 2.08)**	**<0.001**

*HR, hazard ratios; Admission SpO2, peripheral oxygen saturation when inhaling air; AVSD, atrioventricular septal defect; DORV, double outlet right ventricle; TGA, complete transposition of great arteries; CCTGA, congenitally corrected transposition of the great arteries; CPB, cardiopulmonary bypass; VIS, vasoactive-Inotropic score during surgery; TBIL, total bilirubin. Bold values mean statistical p < 0.05*.

### Performance of the PPE Model in the Total Cohort and Different Subgroups

**Figure 1:**
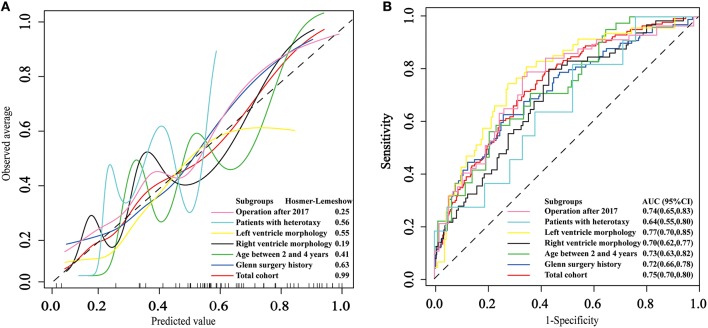
The application and performance of the model in the total cohort and 6 subgroups. **(A)** Calibration curves of the logistic model. **(B)** Receiver operating characteristic curves for prolonged pleural effusion.

The area under ROC curve (AUC) and *p*-value of HL test in the total cohort was 0.75 (0.70, 0.80) and 0.99, respectively. The strongest discrimination ability was achieved in subgroup patients with left ventricle morphology with AUC of 0.77 (0.70, 0.85). The moderate discrimination ability (AUC ≥ 0.7) were also achieved in other 4 subgroups, which included patients with Glenn history, patients whose age between 2 and 4 years, patients diagnosed with right ventricle morphology and patients receiving the operation after 2017. However, poor discrimination (AUC <0.7) were achieved in subgroup patients with heterotaxy syndrome. Good fitness (HL test *p* > 0.05) were found in all six subgroups. The calibration and ROC curves are shown in Figure 1.

### Postoperative Short Term Outcomes

The in-hospital mortality was 2.1% (11/533). The rate of PPE was 27.4% in this study and PPE was associated with in-hospital mortality. Compared to patients without PPE, patients with PPE were associated with longer length of ICU and hospital stay, higher rates of renal replacement treatment and increased hospitalization costs (Table 6).

**Table 6:** Postoperative short-term outcomes in the study patients.

**Variables**	**PE <14d** **(*n* = 381)**	**PE 14-21** **(*n* = 71)**	**PE>21** **(*n* = 73)**	***P*-value**
Reintubation	8 (2.1%)	5 (7.0%)	3 (4.1%)	0.091
Renal replacement treatment	**30 (7.9%)**	**14 (19.7%)**	**11 (15.1%)**	**0.005**
Postoperative length of ICU stay (d)	**4.7 ± 7.0**	**9.2 ± 14.6**	**7.6 ± 13.2**	**0.001**
Postoperative length of hospital stay (d)	**20.8 ± 14.8**	**28.8 ± 14.8**	**46.9 ± 22.2**	**<0.001**
Postoperative costs (thousand RMB)	**102 ± 56.8**	**172 ± 146**	**196 ± 114**	**<0.001**
Total costs (thousand RMB)	**107 ± 57.7**	**177 ± 148**	**203 ± 115**	**<0.001**
In-hospital mortality	**1 (0.3%)**	**2 (2.8%)**	**2 (2.7%)**	**0.025**

*PE, pleural effusion. Bold values mean statistical p < 0.05*.

## Discussions

In our study, we identified 27.4% of all 525 patients as having PPE following TCPC surgery and the independent factors for PPE were age at operation, creation of fenestration, postoperative total protein level, postoperative mechanical ventilation and chylothorax. These predictors were also independent factors for predicting the duration of pleural effusion in Cox regression. The length of hospital stay increased by 18 days and the hospitalization costs increased by 61.7 thousand RMB in patients with PPE after TCPC surgery. Our findings help to stratify high-risk patients and strategies that target the identified risk factors might improve the in-hospital outcomes.

The application of the PPE logistic model to six subgroups were evaluated, because the characteristics of the total cohort were different from those in developed countries, such as age, prior Glenn, the ratio of right ventricle morphology to left ventricle morphology, etc. (2, 3, 11), one of the reasons was that the relatively poorer economic status in our country could lead to the consequences that patients come to the hospital later and receive surgery at a bigger age. In our study, the model had good fitness in the total cohort and six subgroups, and its discrimination ability in the total cohort and subgroups was acceptable. Therefore, the model derived from this study was applicable to not only the Chinese population but also the subgroups, which may be similar to those in other countries.

Different definition for PPE leads to different incidence of PPE. Previous studies had empirically predefined the cutoff value for prolonged duration of pleural effusion as 14 days and the incidence of the PPE was 34.2% (10), which was relatively higher than that in our study (27.4%). However, there is still no consensus on the definition of PPE. In this study, we performed both logistic and Cox regression analysis to free from the adverse effects of dichotomy definition on the results.

No fenestration was an independent factor in our study. Creation of fenestration was recommended as an effective procedure during TCPC operation (14) because fenestration can benefit patients from increased preload of the systemic ventricle if pulmonary blood flow is limited and helps to reduce pulmonary artery pressure if a pulmonary hypertensive crisis occurs. The presence of fenestration was nearly 45.1% in our patients, with a higher rate in more recent years. Our findings were consistent with the study from Zou et al., which found that the creation of fenestration can lower the risk of PPE (9). Overall, fenestration should be performed in patients with high risk for PPE because of its reduction in morbidities and mortality following TCPC surgery (15).

It is noteworthy that a shorter duration of mechanical ventilation predicts lower risk of PPE in our study. Previous studies suggested that early extubation including extubation in operation room is a safe and effective practice following TCPC surgery (16). Furthermore, recent studies demonstrated that prolonged mechanical ventilation after TCPC surgery was associated with adverse in-hospital outcomes, and that early extubation could produce clinical benefits in these patients, such as shorter length of ICU and hospital stay (17, 18). Our data added further evidence to support this opinion by showing that prolonged mechanical ventilation could heighten the risk of PPE and extend length of hospital stay in patients after TCPC surgery. One of the possible explanations behind this finding is that a rise in intra-thoracic pressure and central venous pressure, which is produced by mechanical ventilation, could lead to a decline in lymphatic drainage and reabsorption of pleural fluid (19). In addition, patients with prolonged mechanical ventilation usually require more fluid intake, which might aggravate the PPE. Therefore, maintaining an optimal duration of mechanical ventilation should be one of the prioritized goals in the perioperative management of patients following TCPC surgery.

In our study, young age was an independent risk factor for PPE, which was consistent with the results from Fedderly's study (20). This could be explained by the possible reason that young children are easier to suffer from injuries because the surgery on young children is much more complex than that in adults.

Our findings provide references for both clinical management and future research on complication following TCPC surgery. Our study suggested that optimizing the patents' selection, creation of fenestration in high-risk patients, improving postoperative total protein levels, avoiding prolonged mechanical ventilation and positively dealing with chylothorax can help to lower the risk of PPE in patients after TCPC surgery, shorten length of ICU and hospital stay, and reduce unnecessary hospitalization costs.

## Limitations

There are several limitations in this study. Firstly, this study had a retrospective design. Although a large number of variables were put into the multivariate model to control for the confounders, certain unknown heterogeneities could exist and a patient selection bias may be present due to the non-random design of this study. In addition, the causal association between duration of mechanical ventilation and PPE can not be achieved in this retrospective study. Secondly, perioperative management strategies were to some extent determined by the subjective judgment of the anesthesiologists and the routine practice standards of Fuwai Hospital, so the conclusions drawn from this study could be subject to changes due to the growing experiences of doctors and the modifications of management standards of our hospital. Thirdly, the discrimination power of the model is not strong (AUC < 0.8). Maybe the model did not include other important variables, such as the complexity of the surgery process, which can not be exactly measured, and some catheter parameters like cardiac output, pulmonary vascular resistance, which was measured in some patients in our hospital. Our study could not demonstrate the exact relationship between postoperative infection and PPE. Fourthly, external validation of the model is warranted before its use in clinical practice. Lastly, the lack of data on long-term mortality could limit this study.

## Conclusions

The incidence of PPE in our study was relatively lower than that suggested by previous study and compared to patients without PPE, patients with PPE were associated with a higher incidence of in-hospital mortality. We identified young age, no fenestration, low postoperative total protein, prolonged mechanical ventilation, and chylothorax as independent risk factors to predict PPE. A preventive strategy that targets these factors might reduce the incidence of PPE and shorten length of ICU and hospital stay, and reduce unnecessary hospitalization costs.

## Data Availability Statement

The datasets generated for this study will not be made publicly available Because of data protection policy in our hospital.

## Ethics Statement

This retrospective study was approved by the Institutional Review Boards of Fuwai Hospital, and informed consent was waived because of its retrospective nature.

## Author Contributions

All authors contributed extensively to the work presented in this paper. FY, YJ, and SL proposed the idea of this investigation. QL, WZ, LZ, HW, YLi, and XW were responsible for the collection of data and material. QL helped with the statistical analysis and wrote the manuscript. FY, YJ, ZS, and YLiu helped to revise the manuscript. All authors read and approved the final manuscript.

### Conflict of Interest

The authors declare that the research was conducted in the absence of any commercial or financial relationships that could be construed as a potential conflict of interest.
